# Multivariable Urine Flow Cytometry–Based Screening for Prediction of Urine Culture Positivity

**DOI:** 10.3390/diagnostics16071022

**Published:** 2026-03-28

**Authors:** Darija Knežević, Maja Travar, Đorđe Stojisavljević, Duška Jović, Milorad Grujičić

**Affiliations:** 1 Faculty of Medicine, University of Banja Luka, 78000 Banja Luka, Bosnia and Herzegovina; maja.travar@med.unibl.org (M.T.); duska.jovic@med.unibl.org (D.J.); milorad.grujicic@med.unibl.org (M.G.); 2 University Clinical Centre of the Republic of Srpska, 78000 Banja Luka, Bosnia and Herzegovina; 3 University Computer Center, University of Banja Luka, 78000 Banja Luka, Bosnia and Herzegovina; djordje.stojisavljevic@unibl.org

**Keywords:** urine culture, automated urine flow cytometry, urinary tract infection, screening, cut-off

## Abstract

**Background/Objectives**: Urine samples are the most frequently analyzed specimens in clinical microbiology laboratories. Although urine culture remains the gold standard for diagnosing urinary tract infections, it is time-consuming and resource-intensive. Therefore, reliable screening methods capable of predicting urine culture positivity are needed to optimize laboratory workflow. Automated urine analysis based on flow cytometry enables efficient screening and identification of samples with a low probability of bacterial infection, thereby rationalizing microbiological testing. This study evaluated the usefulness of a multivariable approach to support interpretation of flow cytometry results following the implementation of the Sysmex UF-4000 urine flow cytometer. **Methods**: Routinely collected urine samples from outpatients and hospitalized patients were analyzed using the UF-4000 flow cytometer, with a positivity threshold of ≥100 leukocytes/µL. Urinary parameters were compared between samples with positive and negative cultures. Multivariable logistic regression was applied to identify independent predictors of a positive urine culture. Urinary sediment parameters, including leukocyte, bacterial, fungal, and squamous epithelial cell counts, were assessed as covariates. **Results**: Urine samples with positive cultures showed significantly higher leukocyte counts (median 355.0, IQR 146.5–1429.4) and bacterial counts (median 9805.2, IQR 1134.3–45,011.5). Fungal and squamous epithelial cell counts differed only slightly between groups, although the differences were statistically significant (*p* < 0.001). Leukocyte counts were higher in urine samples from which Gram-negative bacteria were isolated compared with samples containing Gram-positive bacterial isolates (*p* < 0.001). The multivariable model demonstrated the most favorable overall performance, combining high sensitivity with improved specificity and the highest negative predictive value (AUC = 0.927). Optimal cut-off values were 70 leukocytes/µL and 105 bacteria/µL. **Conclusions**: Leukocyte and bacterial counts were the strongest predictors of positive urine culture results. A multivariable model including only these two parameters demonstrated high diagnostic accuracy and may serve as a practical screening tool to identify urine samples with a low probability of bacterial infection. The implementation of this approach could support more efficient use of urine cultures and help optimize laboratory workflow.

## 1. Introduction

Urinary tract infections (UTIs) are the most common bacterial infections in the general population and represent a significant problem in clinical practice. Epidemiological data indicate that over the past three decades there has been an increase in mortality associated with these infections [1]. Due to their high prevalence, tendency to recur, and the possibility of rapid spreading to the upper urinary tract, fast and accurate diagnosis of UTIs is essential. Timely diagnosis enables early initiation of appropriate therapy, reduces the risk of complications such as pyelonephritis and sepsis, and contributes to the rational use of antibiotics and a reduction in antimicrobial resistance [2,3].

The gold standard for UTI diagnosis is urine culture, which identifies the causative pathogen and determines its antibiotic susceptibility. Although urine culture is indispensable for confirming the diagnosis, particularly in complicated and nosocomial infections, it is a time- and resource-intensive method [4,5]. Urine culture also has certain limitations, as it cannot detect fastidious, anaerobic, or slow-growing uropathogens, nor polymicrobial infections. In clinical practice, a discrepancy is often observed between negative urine culture results and persistent symptoms of urinary tract infection, which represents a significant diagnostic and therapeutic challenge in everyday clinical work [6,7].

Given the high incidence of urinary tract infections, urine culture is one of the most frequently requested microbiological tests, which places a substantial burden on microbiology laboratories. A large number of samples, a considerable proportion of which are negative or clinically irrelevant, leads to increased consumption of time, materials, and professional staff resources. Therefore, increasing attention is being paid to the rationalization of the diagnostic process, including the use of rapid screening tests and clearly defined indications for performing urine culture, while retaining its role as the gold standard [8,9].

Fluorescence flow cytometry (FFC) is an analytical method that enables rapid measurement of scattered light and fluorescence emission produced by appropriately illuminated cells. Cells or particles are suspended in a liquid medium and generate signals as they individually pass through a light beam [10].

FFC is gaining increasing importance as a rapid screening method in the diagnosis of urinary tract infections. This method enables automated and rapid quantification of bacteria, leukocytes, and other particles in urine, often within a few minutes. An increasing number of studies show that the application of FFC allows reliable identification of samples with a low probability of infection, thereby reducing the number of unnecessary urine cultures. In this way, the time in which results are obtained is shortened, microbiology laboratories are relieved, and more efficient and rational diagnostics are enabled, while reserving urine culture for clinically relevant and suspicious cases [11,12,13].

Although urine culture remains the gold standard in UTI diagnosis, the use of flow cytometry in screening negative samples allows a significant reduction in the number of unnecessary cultures. Its effectiveness, however, varies depending on clinical populations and the definition of infection; automated analyzers detect both viable and non-viable bacterial particles, which may lead to an overestimation of the number of microorganisms compared with conventional culture [6].

Accordingly, in this study we implemented the Sysmex UF-4000 flow cytometer to assess the diagnostic value of urinary sediment parameters in predicting urine culture positivity and to examine the additional benefit of a multivariate approach, with the aim of providing practical guidelines for the interpretation of flow cytometry results in the clinical microbiology laboratory. To our knowledge, this study is among the first studies from our region to evaluate a simplified multivariable screening model based on leukocyte and bacterial counts obtained by the Sysmex UF-4000 urine flow cytometer for predicting urine culture positivity in a large cohort of both outpatient and hospitalized patients under routine clinical laboratory conditions.

## 2. Materials and Methods

### 2.1. Study Design and Data Collection

This was a prospective study conducted in two phases in the Clinical Microbiology Laboratory of the University Clinical Center of the Republic of Srpska. The first phase of the study covered the period during which the Sysmex UF-4000 flow cytometer (Siemens, Tokyo, Japan) was implemented in our laboratory, from January to March 2024, during which 997 urine samples were collected and analyzed. The cut-off value on the UF-4000 with the optimal positivity threshold determined for leukocytes in urine was set at ≥100/µL. Between January and July 2025 (the second phase of the study), 4840 urine samples collected from hospitalized and outpatient patients were submitted to our laboratory for routine bacterial culture. Urine sampling was performed using the clean-catch midstream first-morning urine technique. Urine samples were collected in sterile culture containers with Vacutainer tubes without preservatives and transported to the microbiology laboratory within 2 h of collection. Samples from pediatric patients, as well as from patients with kidney disease and pregnant women, were labeled as “high-risk samples” and were not included in the study, as they were sent directly for culture. Samples with a volume of <2 mL, according to the requirements of the urine flow cytometer manufacturer, were also excluded from the study. All other samples were initially analyzed on the Sysmex UF-4000, and the decision to perform urine culture was subsequently made according to the leukocyte count.

### 2.2. Laboratory Testing

After receipt in the microbiology laboratory, urine samples entered the testing process within a maximum of 30 min. As part of this diagnostic procedure, urine samples were first analyzed using the UF-4000 to determine the presence of leukocytes, bacteria, fungi, and squamous cells prior to further microbiological processing. If the leukocyte count was ≥100/µL, the samples were inoculated using a standard 1 µL loop onto chromogenic media (BioMérieux, Marcy-l’Étoile, France) for the purpose of microorganism identification and quantification. Plates were incubated in a 5% CO_2_ incubator at 37 °C for 18–24 h. Quantitative results were expressed in CFU/mL, and a culture in which ≥100 colonies of a single species were counted (≥10^5^ CFU/mL) was considered the threshold value for UTI. If the bacterial count was between 10^4^ and 10^5^ CFU/mL, the result was assessed according to the clinical status. Negative results were reported as “urine culture sterile” (no colony growth on inoculated plates) or expressed as <10^3^ CFU/mL. Cultures in which two microbial species were identified with growth < 100 colonies, as well as all cultures with more than two microorganisms, were considered contaminated, and a new urine sample was requested [14,15].

On inoculated plates where >100 colonies of one or two microbial species were observed, further identification was performed based on colony color on chromogenic media, conventional biochemical tests, the Vitek 2 system (bioMérieux, Marcy l’Étoile, France), and/or matrix-assisted laser desorption/ionization time-of-flight mass spectrometry (MALDI-TOF MS, Zhuhai DL Biotech Co., Zhuhai, China).

### 2.3. Statistical Analysis

Statistical analysis was performed to evaluate differences in urine sediment parameters between culture-positive and culture-negative samples and to assess their diagnostic performance for predicting urine culture positivity. Samples with contamination were excluded from comparative analyses, regression modeling, and ROC analyses; only culture-positive (significant growth) and culture-negative samples were analyzed. Continuous variables were summarized as medians with interquartile ranges (IQR) due to non-normal distributions. Comparisons between groups were performed using the Mann–Whitney U test. Associations between continuous variables were examined using Spearman’s rank correlation coefficient.

To identify independent predictors of urine culture positivity, multivariable logistic regression analysis was conducted with urine culture result (positive vs. negative) as the dependent variable. Age and urine sediment parameters (leukocyte count, bacterial count, fungal count, and squamous epithelial cell count) were included as covariates. Due to right-skewed distributions, urine sediment parameters were log10-transformed [log10(x + 1)] prior to inclusion in the regression model. Multicollinearity among predictors was assessed using variance inflation factors (VIF), with values below 2 considered indicative of no relevant multicollinearity. Model results are reported as odds ratios (OR) with corresponding 95% confidence intervals (CI). Model fit was assessed using residual deviance and the Akaike information criterion (AIC).

The discriminative performance of individual urine sediment parameters and the multivariable model was evaluated using receiver operating characteristic (ROC) curve analysis. The area under the ROC curve (AUC) was calculated to quantify diagnostic accuracy. Based on ROC analysis, probability thresholds and corresponding cut-off values prioritizing high sensitivity were explored to assess screening performance. Sensitivity, specificity, positive and negative predictive values, likelihood ratios, and the Youden index were calculated for multiple leukocyte cut-off values. Differences between ROC curves were assessed using DeLong’s test. For the multivariable model, the cut-off represents the predicted probability threshold used to classify samples as culture-positive.

Internal validation of the multivariable logistic regression model was performed using 10-fold cross-validation. The dataset was randomly partitioned into ten subsets of approximately equal size. In each iteration, nine subsets were used for model training and the remaining subset for testing. Predicted probabilities obtained from the held-out folds were combined to construct a cross-validated ROC curve and estimate the cross-validated AUC with 95% confidence intervals.

All statistical tests were two-sided, and a *p*-value < 0.05 was considered statistically significant. Statistical analyses were performed using R (version 4.5.2; packages: pROC, stats, car).

## 3. Results

### 3.1. Descriptive Statistics

There were 1518 (38.78%) male and 2396 (61.22%) female participants. Median age was 64 years (IQR 39–74; range 18–98). A total of 3914 urine specimens were included in the analysis. Specimens that tested positive by the UF-4000 system (*n* = 1282; 32.75%) were subsequently inoculated onto microbiological culture media. Among these, 566 samples (44.15%) yielded positive culture results, while 650 (50.7%) were culture negative, and 66 (5.15%) showed mixed culture.

**Figure 1:**
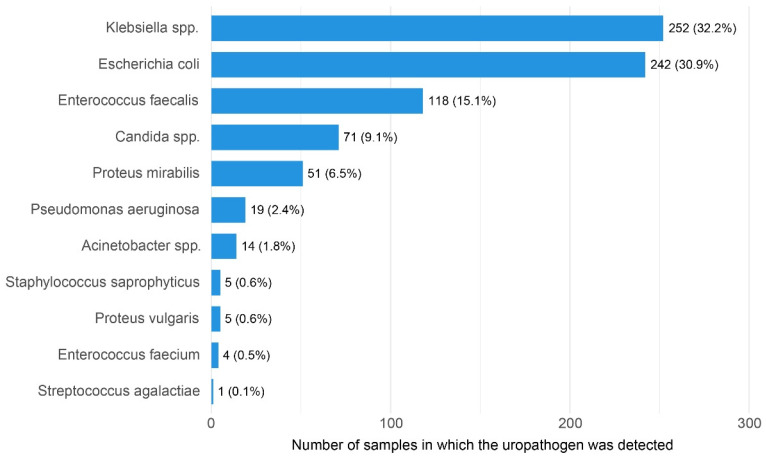
Distribution of uropathogens isolated from positive urine cultures.

The distribution of uropathogens isolated from culture-positive samples is shown in Figure 1.

Urine sediment parameters, including leukocyte count, bacterial count, fungal count, and squamous epithelial cell count, differed between culture-positive and culture-negative samples. A comparative overview of these parameters is presented in Table 1.

As shown in Table 1, culture-positive samples had markedly higher leukocyte and bacterial counts, while fungal counts were modestly higher. Squamous epithelial cell counts showed a small but statistically significant difference between groups. These findings suggest that multiple urine sediment parameters are associated with culture positivity, thus warranting further evaluation in a multivariable model.

### 3.2. Leukocyte Count and Urine Culture Results

**Figure 2:**
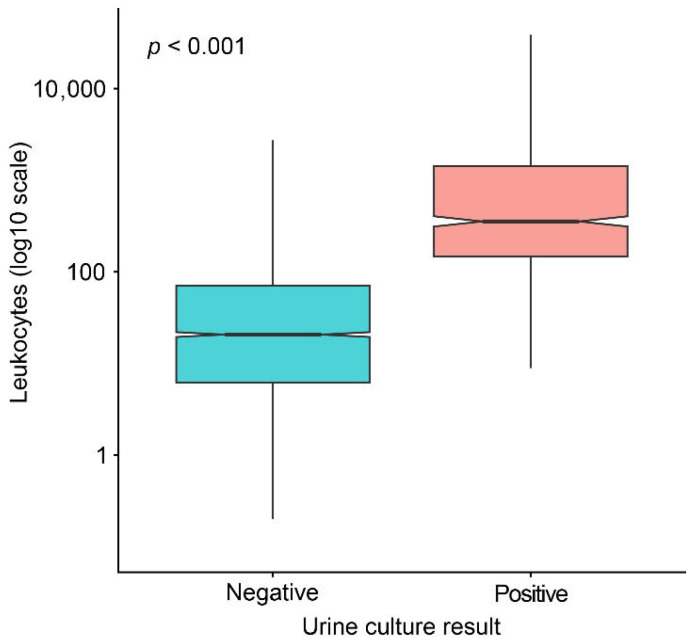
Leukocyte counts vs. urine culture results.

Given the need for a reliable and easily available parameter to guide whether to perform urine culture, the diagnostic value of urine leukocyte count was first examined using basic group separation. Leukocyte counts were compared between samples with positive and negative urine culture results to assess whether this parameter provides sufficient discriminatory information to support its use as a screening marker for culture testing (Figure 2).

Leukocyte counts were significantly higher in samples with positive urine culture results compared with negative samples (Mann–Whitney U test, *p* < 0.001), demonstrating a clear separation between the two groups. This pronounced difference indicates that urine leukocyte count carries relevant diagnostic information and supports its role as a candidate screening parameter for guiding whether to perform urine culture.

### 3.3. Leukocyte Distribution According to Gram Status

**Figure 3:**
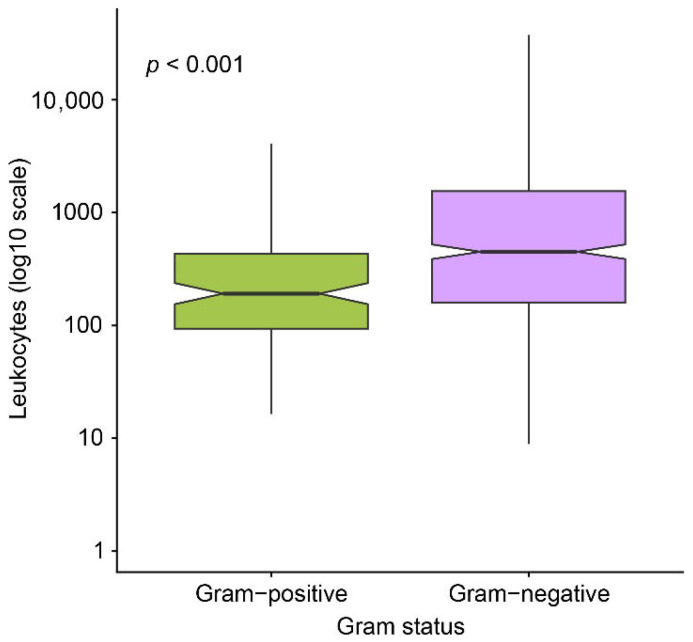
Leukocyte distribution by Gram status.

To further explore the diagnostic relevance of leukocyte counts among culture-positive samples, leukocyte distributions were compared by the Gram status of the isolated bacteria (Figure 3).

Leukocyte counts were higher in Gram-negative infections compared with Gram-positive infections, indicating a stronger inflammatory response associated with Gram-negative uropathogens. This difference was statistically significant, as demonstrated by the Mann–Whitney U test (*p* < 0.001).

### 3.4. Diagnostic Performance and Cut-Off Selection for Leukocyte Count

To evaluate the clinical impact of different leukocyte thresholds for guiding urine culture testing, a range of cut-off values between 10 and 100 leukocytes/µL was systematically assessed using receiver operating characteristic (ROC) analysis. For each cut-off value, sensitivity, specificity, positive and negative predictive values, likelihood ratios, and the Youden index were calculated to characterize diagnostic performance and identify the most appropriate screening threshold (Table 2).

Lower cut-off values were associated with progressively higher sensitivity and lower specificity, reflecting the expected trade-off between detection and false-positive results. The negative likelihood ratio decreased substantially with lower thresholds, indicating improved ability to rule out positive urine cultures. However, reductions below 70 leukocytes/µL resulted in only marginal additional improvement in exclusion performance while causing a pronounced loss of specificity. The cut-off value of 70 leukocytes/µL achieved the highest Youden index, representing the optimal balance between sensitivity and specificity and supporting its use as the most appropriate screening threshold.

**Figure 4:**
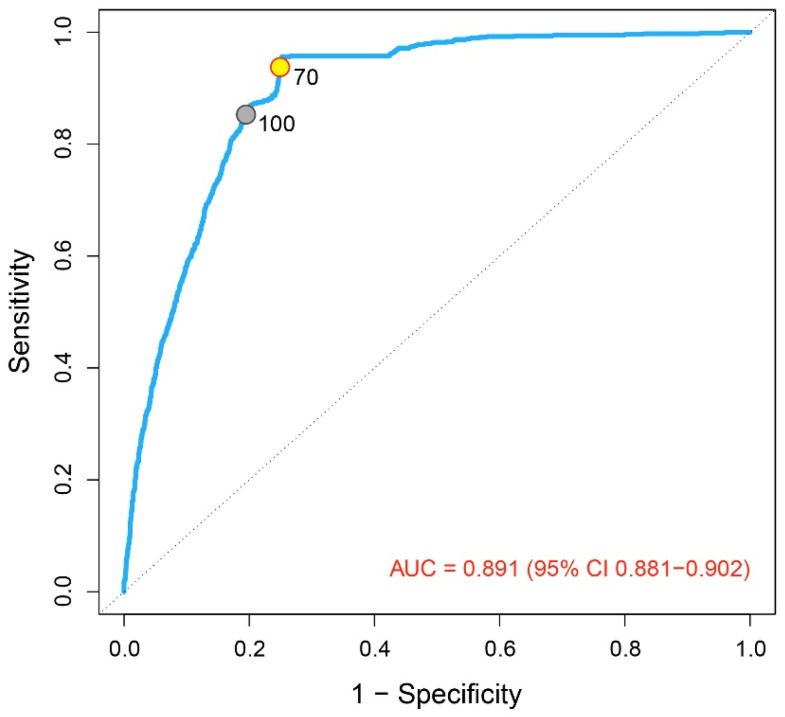
ROC curve for leukocytes (overall; cut-offs: 70 vs. 100 leukocytes/µL).

Receiver operating characteristic (ROC) analysis was performed to assess the overall discriminative ability of urine leukocyte counts for predicting positive urine culture results (Figure 4). The analysis demonstrated excellent diagnostic performance, with an area under the ROC curve (AUC) of 0.891 (95% CI: 0.881–0.902).

The ROC curve illustrates the position of the evaluated cut-off values within the ROC space. Lowering the leukocyte threshold from 100 to 70 leukocytes/µL shifts the operating point toward higher sensitivity with a modest reduction in specificity, consistent with the results presented in Table 2. This visual representation supports the selection of 70 leukocytes/µL as an appropriate screening cut-off, prioritizing the reduction in missed positive cultures.

Among culture-positive samples, lowering the leukocyte cut-off from 100 to 70 leukocytes/µL resulted in a statistically significant increase in sensitivity. McNemar’s test demonstrated a significant difference between the two thresholds (χ^2^ = 64.0, *p* < 0.001), indicating that the cut-off value of 70 leukocytes/µL identified a substantially higher number of culture-positive cases compared with the previously used cut-off of 100.

### 3.5. Multivariable Logistic Regression Analysis

The results of the multivariable logistic regression analysis identifying independent predictors of a positive urine culture are presented in Table 3.

As shown in Table 3, leukocyte count and bacterial count showed the strongest associations with positive urine culture results. Higher leukocyte and bacterial counts were associated with increased odds of culture positivity. Although age was statistically significant, the observed effect size was small and likely reflects residual confounding rather than a clinically meaningful association.

Fungal count was also positively associated with culture positivity, whereas squamous epithelial cell count was inversely associated with the outcome. Increasing age was associated with a small but statistically significant reduction in the odds of a positive urine culture.

The model showed acceptable fit (residual deviance = 2295.9; AIC = 2307.9).

### 3.6. ROC Curve Analysis of Multivariable Model

The discriminative performance of individual urine sediment parameters and the multivariable model was assessed using ROC curve analysis.

**Figure 5:**
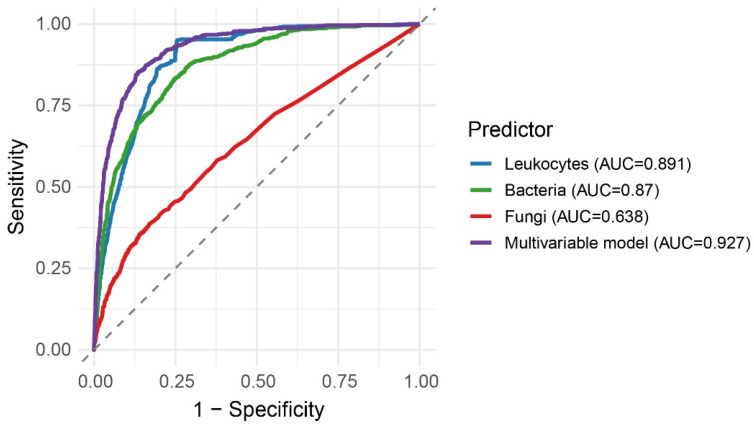
ROC curves illustrating the discriminative performance of individual urine sediment parameters and the multivariable model in predicting urine culture positivity.

ROC curves for leukocyte count, bacterial count, fungal count, and the multivariable logistic regression model are shown in Figure 5.

Among individual urine sediment parameters, leukocyte count showed the highest discriminative ability (AUC = 0.891), followed by bacterial count (AUC = 0.870), whereas fungal count demonstrated limited discriminative performance (AUC = 0.638). The multivariable model outperformed all individual parameters, achieving an AUC of 0.927, indicating excellent overall discrimination between culture-positive and culture-negative samples. Internal validation using 10-fold cross-validation demonstrated stable model performance. The cross-validated AUC was 0.927 (95% CI: 0.918–0.936), confirming the robustness of the multivariable model.

These findings demonstrate improved discrimination when multiple urine sediment parameters are combined.

Based on ROC analysis, selected probability thresholds and corresponding cut-off values are summarized in Table 4. For the multivariable model, the cut-off represents the predicted probability threshold used to classify samples as culture-positive.

All three approaches demonstrated high negative predictive values, indicating their good ability to exclude urine culture positivity. Among single-parameter strategies, leukocyte count achieved a better balance between sensitivity and specificity compared with bacterial count alone.

The multivariable model showed the most favorable overall performance, combining high sensitivity with improved specificity and the highest negative predictive value. These findings demonstrate that combining multiple urine sediment parameters improves diagnostic performance (as shown in Table 4 and Figure 5).

## 4. Discussion

In this study, we evaluated the diagnostic performance of urine sediment parameters obtained by automated urine flow cytometry for predicting urine culture positivity and assessed the added value of a multivariable approach. Our findings demonstrate that leukocyte count and bacterial count are the strongest individual predictors of a positive urine culture, while a multivariable logistic regression model combining several urine sediment parameters offers superior discriminative performance. These results are in close agreement with previously published studies evaluating flow cytometry–based screening strategies for urinary tract infection (UTI) [13,16,17,18].

Consistent with earlier investigations, leukocyte and bacterial counts showed strong associations with culture positivity and high discriminative ability. Previous studies using Sysmex UF-series analyzers have repeatedly reported that bacterial counts measured by flow cytometry are highly sensitive for identifying culture-positive samples and particularly effective for ruling out negative cultures, supporting their use as a screening tool rather than a definitive diagnostic method [19,20,21]. Similarly, leukocyte counts have been shown to contribute substantially to UTI prediction, with reported AUC values comparable to those observed in the present study. Our results corroborate these findings, demonstrating AUC values close to 0.90 for both leukocytes and bacteria, placing their performance at the upper range of values reported in the literature [22,23].

These findings may be partly explained by the distribution of uropathogens observed in the present study. The distribution of uropathogens was consistent with the known epidemiology of urinary tract infections, with Gram-negative bacteria predominating. *Escherichia coli* and *Klebsiella* spp. accounted for the majority of positive cultures, which likely contributed to the strong discriminative performance of urine sediment markers, particularly leukocyte and bacterial counts, observed in the ROC analyses. Gram-positive pathogens, primarily *Enterococcus* spp., were less frequently identified, while fungal isolates, mainly *Candida* spp., represented a smaller but clinically relevant proportion of positive cultures. The relatively low prevalence of other uropathogens, such as *Pseudomonas aeruginosa* and *Acinetobacter* spp., reflects their typical association with specific patient populations and clinical settings rather than the general study cohort.

Importantly, our multivariable logistic regression model outperformed all individual urine sediment parameters, achieving excellent overall discrimination between culture-positive and culture-negative samples. Internal validation using 10-fold cross-validation confirmed that the model maintained stable discriminative performance, with a cross-validated AUC identical to that observed in the primary ROC analysis. This finding suggests that the predictive performance of the model is unlikely to be driven by overfitting and supports its potential applicability in routine laboratory practice. Although internal validation confirmed the robustness of the model, external validation in independent cohorts will be necessary to assess its generalizability across different clinical settings.

Although several urinary sediment parameters were initially evaluated in the multivariable analysis, only leukocyte and bacterial counts remained significant predictors of urine culture positivity in the final model. This finding is consistent with the biological basis of urinary tract infections, where bacteriuria and the associated inflammatory response reflected by leukocyturia represent the most relevant indicators of infection. In contrast, fungal elements and squamous epithelial cells showed statistically significant differences between groups but did not provide additional predictive value when included in the multivariable model. Therefore, the final model retained only leukocyte and bacterial counts, resulting in a simpler and clinically applicable approach for predicting urine culture positivity. This observation aligns with a growing body of evidence indicating that combining multiple urine flow cytometry parameters improves diagnostic accuracy compared with single-marker strategies. Recent studies employing multivariable regression models or composite risk scores have shown that integrating leukocytes, bacterial counts, and additional urine sediment features yields superior discrimination and more robust screening performance [24,25]. While some authors have applied complex machine learning techniques, our findings demonstrate that a transparent and interpretable multivariable logistic regression model can achieve comparable or superior performance, which is particularly advantageous for clinical implementation and routine laboratory use.

A noteworthy finding of this study is the inverse association between squamous epithelial cell count and urine culture positivity. Squamous epithelial cells are commonly regarded as indicators of sample contamination, and their presence has been linked to mixed or non-significant bacterial growth in previous reports [26]. Our results quantitatively confirm this relationship by demonstrating an independent negative association with culture positivity after adjustment for other urine sediment parameters. This finding supports the inclusion of squamous epithelial cell count as a relevant parameter in predictive models and underscores its potential value in assessing sample quality for routine practice.

The ROC-based cut-off analysis further supports the clinical utility of urine flow cytometry as a screening tool. The selected cut-off values achieved high sensitivity and very high negative predictive values, indicating that samples below the proposed thresholds are highly unlikely to yield urine culture positivity. In addition to sensitivity and negative predictive value, the likelihood ratio for a negative result (LR−) was very low, further supporting the strong ability of the proposed screening strategy to exclude urine culture positivity. This finding reinforces the suitability of the model for screening purposes, where safely ruling out infection is of primary importance. This observation is consistent with previous studies emphasizing that the principal benefit of automated urine flow cytometry lies in safely reducing unnecessary urine cultures while maintaining a low risk of missing clinically relevant infections. As highlighted in earlier reports, optimal cut-off values may vary depending on the patient population and clinical setting, underscoring the importance of local validation rather than applying universal thresholds [27,28].

Taken together, the results of the present study reinforce the role of automated urine flow cytometry as an effective screening approach for urinary tract infection. Our findings confirm that leukocyte and bacterial counts are key predictors of culture positivity and demonstrate that a multivariable model incorporating additional urine sediment parameters provides superior diagnostic performance. The observed inverse association with squamous epithelial cells further supports their relevance as a marker of sample quality. Overall, these results are consistent with existing literature and provide additional evidence supporting the integration of multivariable urine sediment analysis into routine laboratory workflows to improve screening efficiency and diagnostic accuracy.

The interpretation of leukocyturia must be approached with caution, particularly in cases where increased white blood cell counts are not accompanied by positive urine cultures. Pyuria in the absence of bacteriological growth, commonly referred to as sterile pyuria, represents a frequent finding and highlights the limited specificity of leukocyturia as an isolated marker of urinary tract infection. This observation is especially relevant in the context of automated urine flow cytometry, where leukocyte counts may be elevated despite the absence of clinically significant bacteriuria [16,19]. Sterile pyuria may arise from a variety of infectious and non-infectious conditions, including prior antibiotic exposure and infections caused by fastidious or atypical microorganisms not detected by routine cultures, as well as inflammatory or structural disorders of the urinary tract. Consequently, the presence of leukocyturia alone does not necessarily indicate a bacterial infection requiring antimicrobial therapy. These findings reinforce the importance of integrating leukocyte parameters with bacterial counts and clinical data rather than relying on leukocyturia as a sole screening criterion [29].

Taken together, these findings align with previous reports suggesting that the principal benefit of automated urine flow cytometry lies in its capacity to reduce unnecessary microbiological testing while maintaining a low risk of missing clinically relevant infections. However, the presence of leukocyturia without bacteriuria may help exclude bacterial infection and reduce unnecessary microbiological cultures and antimicrobial therapy. Furthermore, the variability in the prevalence and causes of sterile pyuria across different patient populations and clinical settings underscores the importance of local validation of cut-off values rather than applying universal thresholds [30,31].

The clinical implications of our findings relate to the potential use of urinary flow cytometry as a screening tool to optimize urine culture testing. The multivariable model combining leukocyte and bacterial counts showed a high negative predictive value, indicating that samples classified as negative have a very low probability of yielding a positive culture. However, maintaining a very low false-negative rate remains essential to minimize the risk of missing clinically significant infections. In routine clinical microbiology laboratories, where urine samples represent a large proportion of submitted specimens, even a moderate reduction in culture requests may result in a significant decrease in laboratory workload and associated costs. The application of automated urine flow cytometry using the Sysmex UF-4000 may therefore contribute to a more efficient use of laboratory resources and shorter turnaround times.

A limitation of this study is the lack of detailed clinical data regarding underlying urological or gynecological conditions. Therefore, we were unable to perform subgroup analyses to evaluate whether specific urinary or gynecological diseases influence flow cytometry parameters. Future studies including well-characterized clinical populations could further clarify the diagnostic performance of this approach in different patient groups.

## 5. Conclusions

This study demonstrates that urine sediment parameters obtained by automated urine flow cytometry are valuable predictors of urine culture positivity and can be effectively used for screening purposes. Leukocyte and bacterial counts emerged as the strongest individual predictors, showing high discriminative performance, while fungal counts and squamous epithelial cell counts provided additional, complementary information.

Importantly, a multivariable logistic regression model integrating multiple urine sediment parameters outperformed individual markers and achieved excellent overall discrimination between culture-positive and culture-negative samples. These findings complement the high sensitivity and negative predictive value observed for the selected cut-off values, supporting the concept that combining several flow cytometry–derived parameters enhances diagnostic accuracy compared with single-parameter approaches.

The selected cut-off values yielded high sensitivity and very high negative predictive values, indicating that the proposed approach is particularly useful for ruling out urine culture positivity rather than identifying positive cultures, thereby helping to reduce unnecessary urine cultures in routine practice. The inverse association observed for squamous epithelial cells further emphasizes their role as an indicator of sample quality rather than true infection.

Sterile pyuria should be regarded as a non-specific marker of inflammation, requiring interpretation in accordance with the clinical picture and complementary laboratory data. This is particularly important in automated urine screening algorithms, where the presence of leukocytes in the absence of concurrent bacteriuria can help safely rule out bacterial infection and reduce unnecessary microbiological testing.

Overall, these results are consistent with previously published literature and reinforce the role of automated urine flow cytometry as an efficient and clinically meaningful screening tool for urinary tract infection. The implementation of multivariable urine sediment analysis may improve laboratory workflows and support timely clinical decision-making, while local validation remains essential to optimize performance across different patient populations.

## Figures and Tables

**Table 1:** Urine sediment parameters by urine culture result.

Parameter	Positive (Median, IQR)	Negative (Median, IQR)	*p*-Value
Leukocytes	355.0 (146.5–1429.4)	20.6 (6.1–70.8)	<0.001
Bacteria	9805.2 (1134.3–45,011.5)	113.5 (27.5–566.7)	<0.001
Fungi	2.0 (0.5–112.1)	0.8 (0.2–2.5)	<0.001
Squamous epithelial cells	3.8 (1.1–12.4)	3.1 (0.8–10.8)	<0.001

**Table 2:** Diagnostic accuracy measures for urine leukocyte count across multiple cut-off values for predicting positive urine culture results.

Cut-Off	Sensitivity	Specificity	PPV	NPV	LR+	LR−	Youden
10	0.994	0.340	0.224	0.996	1.505	0.019	0.333
20	0.982	0.490	0.270	0.993	1.927	0.037	0.472
30	0.958	0.584	0.307	0.986	2.302	0.072	0.542
40	0.958	0.646	0.342	0.988	2.709	0.065	0.604
50	0.958	0.685	0.369	0.988	3.044	0.062	0.643
60	0.958	0.721	0.398	0.989	3.437	0.059	0.679
70	0.954	0.748	0.421	0.988	3.781	0.062	0.702
80	0.878	0.771	0.424	0.971	3.829	0.158	0.649
90	0.873	0.791	0.445	0.970	4.174	0.160	0.664
100	0.862	0.806	0.460	0.968	4.432	0.172	0.667

PPV—Positive Predictive Value; NPV—Negative Predictive Value; LR(+)—Positive Likelihood Ratio; LR(−)—Negative Likelihood Ratio.

**Table 3:** Multivariable logistic regression results predicting positive urine culture (dependent variable: positive urine culture result).

Predictor	Odds Ratio (OR)	95% CI	*p*-Value
Age (years)	0.99	0.98–0.99	<0.001
Log_10_(Leukocytes + 1)	3.18	2.73–3.72	<0.001
Log_10_(Bacteria + 1)	3.45	3.07–3.88	<0.001
Log_10_(Fungi + 1)	1.37	1.21–1.54	<0.001
Log_10_(Squamous epithelial cells + 1)	0.37	0.29–0.46	<0.001

**Table 4:** Diagnostic performance of selected cut-off values for urine culture screening.

Predictor	Cut-Off	Sensitivity (%)	Specificity (%)	PPV (%)	NPV (%)
Leukocytes	70.0	92.5	74.8	41.5	98.1
Bacteria	105.0	95.0	48.7	25.8	98.1
Multivariable model	P ^1)^ ≥ 0.058	95.0	70.6	37.7	99.0

^1)^ P denotes predicted probability from the multivariable model. PPV—Positive Predictive Value; NPV—Negative Predictive Value.

## Data Availability

Data supporting the findings of this study are available from the corresponding author upon reasonable request.
